# Increased violence and aggression levels during the SARS-Cov-2 pandemic; data from three London acute psychiatric inpatient facilities.

**DOI:** 10.1192/j.eurpsy.2023.1924

**Published:** 2023-07-19

**Authors:** S. Bonaccorso, O. Ajnakina, A. Ricciardi, S. Ouabbou, J. Wilson, C. Theleritis, M. Badhan, A. Metastasio, N. Stewart, M. Barczyck, F. Johansson, T. Tharmaraja, F. Schifano

**Affiliations:** 1 UCL; 2 Camden & Islington NHS Foundation Trust; 3 King’s College London, London, United Kingdom; 4 Dept of Mental Health ASL, Rome, Italy; 5 University of Athens, 1st Psychiatry Dept, Athens, Greece; 6 University of Hertfordshire, Hatfield, United Kingdom

## Abstract

**Introduction:**

The COVID-19 pandemic has significantly impacted mental health services, with the literature reporting an increase in the incidence of psychiatric admissions.

**Objectives:**

The aim of this study was to assess the impact of the pandemic on clinical presentations, characteristics of admission and incidents occurring in three acute inpatient mental health facilities in the UK.

**Methods:**

This was a retrospective study comparing data from the first and third UK lockdown to the five years prior to the pandemic. Data was acquired from electronic clinical records and addressed two acute psychiatric inpatient wards and one psychiatric intensive care unit. Key outcomes of comparison were clinical presentations, number of admissions, length of hospital stay, number of incidents and characteristics of incidents.

**Results:**

Compared to the previous 5 years, a higher number of incidents characterized by violence and aggression were reported during the first (56.8% vs 44.3%, x^2^=16.56, df=1, p<0.001) and third lockdown (100.0% vs 86.2%, x^2^=36.40, df=1, p<0.001). An increase in non-psychotic disorders was observed in the first lockdown (20.0% vs 13.1%, *x*^2^=4.76, df=1, p=0.029), whilst increased first episode psychosis (19.7% vs 11.3%, *x*^2^=8.1, df=1, p=0.004) and schizophrenia spectrum disorders (74.4% vs 57.2%, *x*^2^= 7.6, df=1, p=0.006) were diagnosed during the third lockdown. There were no significant changes in the diagnosis of mood disorders in both lockdowns compared to previously. The median length of inpatient stay significantly reduced during the first lockdown (28 days vs 36 days, *x*^2^= 7.66, df=1, p=0.006).

**Conclusions:**

Increased inpatient incidents may be explained by the impact of the pandemic on staffing levels and resources, combined with increased emotional distress amongst patients in the face of uncertainty. The pandemic may have increased substance misuse potentially linked with the increased incidence of first episode psychosis.

**Disclosure of Interest:**

S. Bonaccorso: None Declared, O. Ajnakina: None Declared, A. Ricciardi: None Declared, S. Ouabbou: None Declared, J. Wilson: None Declared, C. Theleritis: None Declared, M. Badhan: None Declared, A. Metastasio: None Declared, N. Stewart: None Declared, M. Barczyck: None Declared, F. Johansson: None Declared, T. Tharmaraja: None Declared, F. Schifano Speakers bureau of: Prof. Fabrizio Schifano is a member of the European Medical Agency

